# Emerging natural and tailored perovskite-type mixed oxides–based catalysts for CO_2_ conversions

**DOI:** 10.3389/fchem.2022.961355

**Published:** 2022-08-05

**Authors:** Juan Wu, Runping Ye, Dong-Jie Xu, Lingzhong Wan, Tomas Ramirez Reina, Hui Sun, Ying Ni, Zhang-Feng Zhou, Xiaonan Deng

**Affiliations:** ^1^ Institute of Cotton, Anhui Academy of Agricultural Sciences, Hefei, China; ^2^ Key Laboratory of Jiangxi Province for Environment and Energy Catalysis, Institute of Applied Chemistry, School of Chemistry and Chemical Engineering, Nanchang University, Nanchang, China; ^3^ Key Laboratory of Coal to Ethylene Glycol and Its Related Technology, Fujian Institute of Research on the Structure of Matter, Chinese Academy of Sciences, Fuzhou, China; ^4^ Department of Chemical and Process Engineering, University of Surrey, Guildford, United Kingdom; ^5^ Department of Inorganic Chemistry and Materials Sciences Institute, University of Seville-CSIC, Seville, Spain

**Keywords:** CO_2_ conversions, oxygen vacancies, dispersion of active metal, strong metal-support interactions, perovskite-type mixed oxide based catalysts

## Abstract

The rapid economic and societal development have led to unprecedented energy demand and consumption resulting in the harmful emission of pollutants. Hence, the conversion of greenhouse gases into valuable chemicals and fuels has become an urgent challenge for the scientific community. In recent decades, perovskite-type mixed oxide-based catalysts have attracted significant attention as efficient CO_2_ conversion catalysts due to the characteristics of both reversible oxygen storage capacity and stable structure compared to traditional oxide-supported catalysts. In this review, we hand over a comprehensive overview of the research for CO_2_ conversion by these emerging perovskite-type mixed oxide-based catalysts. Three main CO_2_ conversions, namely reverse water gas shift reaction, CO_2_ methanation, and CO_2_ reforming of methane have been introduced over perovskite-type mixed oxide-based catalysts and their reaction mechanisms. Different approaches for promoting activity and resisting carbon deposition have also been discussed, involving increased oxygen vacancies, enhanced dispersion of active metal, and fine-tuning strong metal-support interactions. Finally, the current challenges are mooted, and we have proposed future research prospects in this field to inspire more sensational breakthroughs in the material and environment fields.

## Introduction

The rapid development of society and the economy has led to the huge demand for global energy (Vignieri, 2020). Although renewable energy resources such as tidal, geothermal power, wind, and solar have emerged in recent years, traditional fossil fuels including coal, oil, and natural gas are still dominant within the energy portfolio (Li 2021; Zhao et al., 2021). The high reliance on fossil fuels is accompanied by massive greenhouse gases (GHGs) emissions, mostly in the form of carbon dioxide (CO_2_), which brews a potential threat to the ecological environment and human health (Roy et al., 2018). According to the World Energy Statistical Yearbook (70th Edition) released by the British Petroleum Company, global carbon emissions have maintained continuous growth since 2013 and reached a formidable record of 3.436 × 10^10^ tons in 2019 (Hu et al., 2021). As a result, a series of global action plans such as the Intergovernmental Panel on Climate Change (IPCC), the United Nations Climate Change Conference (COP21, Paris, 2015), and the International Energy Agency (IEA) have accentuated the imperativeness to diminish CO_2_ emissions by at least half of the current amount by 2050 (Roy et al., 2018; Hussain et al., 2021). China has come up with the target to reach a “carbon peak” by 2030 and be “carbon neutral” by 2060 in carbon dioxide emissions (Wang et al., 2020; Li 2021; Zhao et al., 2021). Therefore, the conversion and utilization of waste CO_2_ emissions into higher-value commodities while mitigating climate change has drawn great attention, which is critical for a sustainable future (Ye et al., 2019a; Sun et al., 2020; Ye et al., 2020).

However, CO_2_ is a highly oxidized, thermodynamically stable molecule (∆G^0^ = -400 kJ/mol) with ultra-low reactivity, which requires surmounting the tremendous thermodynamic activation barrier. Thus, the chemical conversion and economic utilization of CO_2_ is an awesome scientific and technical challenge (Ashok et al., 2020). CO_2_ is mainly used as raw material to manufacture fuels or bulk chemicals for the chemical industry in the following ways: 1) CO_2_ to CO (Chen et al., 2020; Kopac et al., 2020; Lim et al., 2021b); 2) CO_2_ to CH_4_ (Shin et al., 2016; Ulmer et al., 2019; Wang X et al., 2019); 3) CO_2_ to CH_3_OH (Zhan et al., 2014; Li et al., 2017; Li F et al., 2019); 4) CO_2_ to bulk chemicals like DME, urea, salicylic acid, polycarbonates (Utsis et al., 2016; Ye et al., 2019a; An et al., 2021). Among the proposed CO_2_ recycling options, catalytic CO_2_ hydrogenation to carbon fuels, especially *via* CO as an intermediate for the Fischer-Tropsch synthesis to generate more complicated chemicals, is of particular industrial importance (Gao et al., 2020). Thence, hydrogenation reaction has been regarded as an influential chemical conversion of CO_2_ since it offers a promising prospect to achieve sustainable development in energy and the environment. However, CO_2_ hydrogenation and conversion technology are still challenging for inadequate conversion and poor selectivity, which are outcomes of unfavorable kinetic and thermodynamic factors (Moradi et al., 2010). For example, CO_2_ conversion involves selective reduction of CO_2_ with H_2_ or another reductant under high temperatures and pressures, while metal-based catalysts used are inclined to sinter and deactivate under severe operating conditions, thus the use of improved catalysts or an alternative approach is necessary (Tavasoli and Ozin 2018). During the reaction, carbon deposition on the surface of the catalyst is the most frequent reason for catalyst deactivation because the access of reactant molecules to the active metal sites was hampered (Li and Gong 2014). Thence, the solution to these issues is to develop catalysts and integrated reactor systems with high efficiency and specific selectivity to produce products with high conversion and minimal energy consumption among industrial time scales (Rodriguez et al., 2017; Liu et al., 2020a).

Among the various materials, the perovskites-type mixed oxides-based catalysts have become the focus of research due to their high-temperature thermochemical stability and high oxygen transport properties (Huang et al., 2018). Compared with traditionally supported catalysts, most of the active metals are substituted in the crystal structure and only a small fraction of active metals is on the surface in perovskites-type mixed oxides-based catalysts (Zhu et al., 2014; Wu et al., 2018). The substituted active metal particles would be exsolved to the surface under reduction atmosphere to gain highly dispersed metal crystals on the surface, which performed outstanding resistance to coarsening and agglomeration (Messaoudi et al., 2018). These inherent properties allow perovskite-type mixed oxides-based catalysts to have a wide range of applications in chemical catalysis (Ishikawa et al., 2020; Wang K et al., 2021; Zhu and Thomas 2009), electrochemical catalysis (Okamoto and Suzuki 2014; Yin et al., 2019), and photocatalysis (Peng et al., 2020; Xu R et al., 2020). As for the structural properties of perovskite-type mixed oxides-based catalysts, we will describe them in detail in the second section of this review.

CO_2_ hydrogenation and conversion technology need high temperatures to ensure thermodynamically favorable conditions, and naturally, lots of people have applied perovskite-type catalysts in this process (Su et al., 2016). Under the high reducing temperatures, the perovskite oxides are recognized to be partly reduced, leading to the formation of nanoparticles of B site metals, which are not only active for the reforming reaction but also insusceptible to carbon deposition (Jing et al., 2009; Tsiotsias et al., 2020). For example, le Saché et al. have applied a La_2_Zr_2-x_Ni_x_O_7-δ_ pyrochlore-double perovskite catalyst for gas-phase CO_2_ recycling conversion, and the active Ni clusters were exsolved from pyrochlore-double perovskite materials after the reaction leading to highly dispersed active ensembles which account for the high activity and stability of the catalyst during CO_2_ recycling conversion reactions (le Saché et al., 2020). Valderrama et al. synthesized a series of perovskite-type oxides based on La-Sr-Co (La_1-x_Sr_x_CoO_3_) used as precursors for the catalytic CO_2_ reforming of CH_4_, the Co^0^ nano-size particles are achieved and highly dispersed in the La_2_O_2_CO_3_-SrO solid matrix after activation/reduction process which leading to high activity performance (Valderrama et al., 2013). Perovskites-type based materials with a defined element have been reviewed for specific CO_2_ conversion reactions (Tabish et al., 2020; Madi et al., 2021), as far as we know, the review on the perovskites-type based catalysts for the thermal CO_2_ conversions has been rarely reported. . Here, we have especially attempted to expatiate on the catalytic pathways and the position of perovskite-type mixed oxides based catalysts in deciding the selectivity of CO_2_ hydrogenation and conversion to CO and CH_4_. In particular, we classify the main reactions for catalytic CO_2_ hydrogenation and conversion: 1) reverse water gas shift reaction (RWGS), 2) CO_2_ methanation, and 3) CO_2_ reforming of methane. We would provide an elaborated account of recent perovskite-type mixed oxides-based catalyst developments, together with the pathways and mechanisms of reactions. In addition to showing the latest optimal catalysts including their properties, we also provide the challenges that need to be dealt with and prospects for future research and development.

## Perovskites-type mixed oxides-based catalysts

The performance of a catalyst largely depends on the structural and geometric parameters of the surface (Monteiro et al., 2019; Kopac et al., 2020; Riani et al., 2021). Apart from the traditionally supported catalysts, a class of crystalline oxide catalysts has attracted extensive attention due to their excellent thermal stability (Godding et al., 2019; Koch et al., 2020). In these materials, the active sites are incorporated into the structure, resulting in catalysts that are thermally stable at high temperatures. Moreover, a few of them possess instinctive oxygen mobility that can be strengthened by the replacement of active metals in the lattice, which is helpful to mitigate carbon deposition (Li M et al., 2020; Peng et al., 2020). Large numbers of these materials, such as perovskites (Huang et al., 2018; Ishikawa et al., 2020; Koch et al., 2020), pyrochlores (Li et al., 2016; Talanov and Talanov 2021; Trump et al., 2018; le Saché et al., 2018), fluorites (Chen et al., 2019; Gao et al., 2021), and hexa-aluminates (Tian et al., 2016; Xu L et al., 2020) have been investigated for varied high-temperature reactions.

**FIGURE 1:**
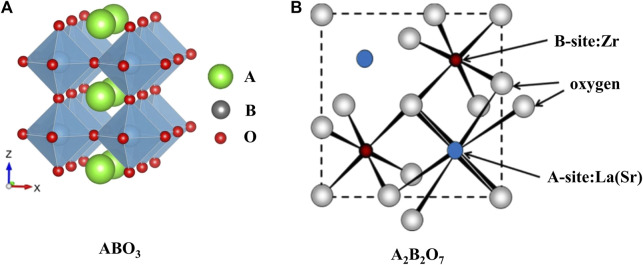
Ideal models of ABO_3_ **(A)** ((Ji et al., 2020); Copyright ^©^ 2020 (Royal Society of Chemistry) and A_2_B_2_O_7_ **(B)** [(Pakhare and Spivey 2014); Copyright ^©^ 2014 (Royal Society of Chemistry)] structure.

Perovskite-type oxides (ABO_3_), which acquire the structure that large cation A locates on the edge and smaller cation B locating in the center of the octahedron, as shown in Figure 1A (Ji et al., 2020), are favorable materials to catalyze high-temperature reactions due to their tunable catalytic properties and thermal stabilities. Generally, the A site is filled with lanthanide metals (La, Nd, Sm, etc.) or alkaline earth metals (Sr, Ca, etc.), and the B site element is chosen from the transition metals (Fe, Ni, Mn, etc.) (Shin et al., 2016; Mateo et al., 2021). Another class of crystalline oxide materials with the general formula A_2_B_2_O_7_ has been used for CO_2_ conversion reactions (Kumar et al., 2016; Fang et al., 2021). The metals of the framework are similar to those of perovskite-type mixed oxides (ABO_3_) based materials and its model, as shown in Figure 1B. Namely, the larger rare-earth trivalent metal like La is at the A position, and the smaller tetravalent transition metal like Zr, Ti occupies the B site of these materials. However, the formation of the crystal phase depends on the ionic radius ratio of A-position to B-position: when the ratio is over 1.8, a perovskite structure appears; if the ratio is in the range of 1.4–1.8, pyrochlore is the dominant structure; and the fluorite phase prevails when the ratio is less than 1.4 (Pakhare and Spivey 2014).

The crystalline oxides can be prepared using the Pechini sol-gel method (Haynes et al., 2008; Haynes et al., 2009; Blanco et al., 2022). Ethylene glycol or citric acid are used as complexing materials to mingle with the metal precursors (Li S et al., 2020; Onrubia-Calvo et al., 2021). The resulting amorphous resins, which are precursors of the perovskites, pyrochlores, or fluorites, are then calcined at high temperatures (usually 800–1000°C) to remove the remaining organics and construct the crystallographic ABO_3_ or A_2_B_2_O_7_ phase (Haynes et al., 2009; Kumar et al., 2016). The catalytic activity of ABO_3_ or A_2_B_2_O_7_ can be modulated by partial replacement of cations at the A and/or B sites, leading to the formation of structural defects to stabilize the uncommon oxidation states by B site components (Hare et al., 2018; Jiang et al., 2021; Zhang J et al., 2021). The appealing properties of crystalline oxides for catalytic reactions involve the high oxygen movability and stability of uncommon oxidation states in the structure, as well as high-temperature thermal stability (Su et al., 2014; Bai et al., 2019). In both pyrochlores and perovskites, most of the active metals are replaced inside the body of the crystal structure, except for a small percentage at the surface (Moradi et al., 2012; Bhavani et al., 2013; Valderrama et al., 2018). Under a reducing atmosphere, the transition metals could be exsolved to the surface of oxide to form highly dispersed crystals, meanwhile, the reduced states can be used as supported catalysts too (Valderrama et al., 2005; Valderrama et al., 2013; Yang et al., 2018). As the exsolution method can immobilize the particles more firmly on the support than the impregnation method, the exsolved particles have outstanding insusceptibility to coarsening and agglomeration. Therefore, the catalytic activity of the exsolved particles is more stable during the reaction operation. Moreover, the highly dispersed particles inhibit the formation of carbon deposition, thusly preventing the deactivation of catalysts (Jing et al., 2009; Wang X et al., 2019; Lim et al., 2021a).

## Perovskites-type mixed oxides-based catalysts applied in CO_2_ conversion

We discuss the CO_2_ conversions, namely 1) reverse water gas shift reaction (RWGS), 2) CO_2_ methanation reaction, and 3) CO_2_ reforming of methane to form target products mainly over perovskites-type mixed oxides based catalysts. Before discussing the reaction performance of the crystalline oxide catalyst, we first briefly introduce the CO_2_ conversion reactions. Subsequently, we introduce the application of perovskite-type mixed oxides-based catalysts in CO_2_ conversion reactions, especially regarding the modification of perovskite with improving the reaction performance. Finally, we give an outlook on the future application of perovskite catalysts in CO_2_ conversions.

### RWGS reaction

The hydrogenation of CO_2_ to CO, commonly referred to as the reverse water gas shift reaction (RWGS), is one of the most technically achievable reactions to realize the clean utilization of CO_2_ as an abundant renewable carbon source (Chen et al., 2020; Wang X et al., 2021). Apart from generating CO, this reaction may also be regarded as an intermediate process (e.g., CO_2_ methanation) for supplementary fuel and chemical synthesis (Hare et al., 2019a). The RWGS reaction is a reversible and energy-intensive way (Eq. 1), and its conversion of CO_2_ and selectivity of CO are typically determined by thermodynamic equilibrium (Liu et al., 2020b).

$$CO_{2}+H_{2}\to \mathrm{CO}+H_{2}\mathrm{O,\Delta Η=41}\mathrm{.5kJ}\;\;\;\mathrm{mo}l^{\mathrm{-1}}$$ (1)

Owing to its endothermic property, the RWGS reaction is typically operated at high temperatures (up to 700 K) to achieve a satisfactory CO_2_ conversion (Liu et al., 2022). However, it could suffer the effect of catalyst sintering deactivation at elevated temperatures. Therefore, improving the catalytic activity at lower temperatures or adopting catalysts with higher temperature stability is the main strategy to realize the industrialization of the RWGS reaction (Kopac et al., 2020; Yang et al., 2020; Lim et al., 2021b; Jo et al., 2022). In any case, green hydrogen is needed for the RWGS when this process is envisaged as a greenhouse gas conversion route (Nityashree et al., 2020).

**FIGURE 2:**
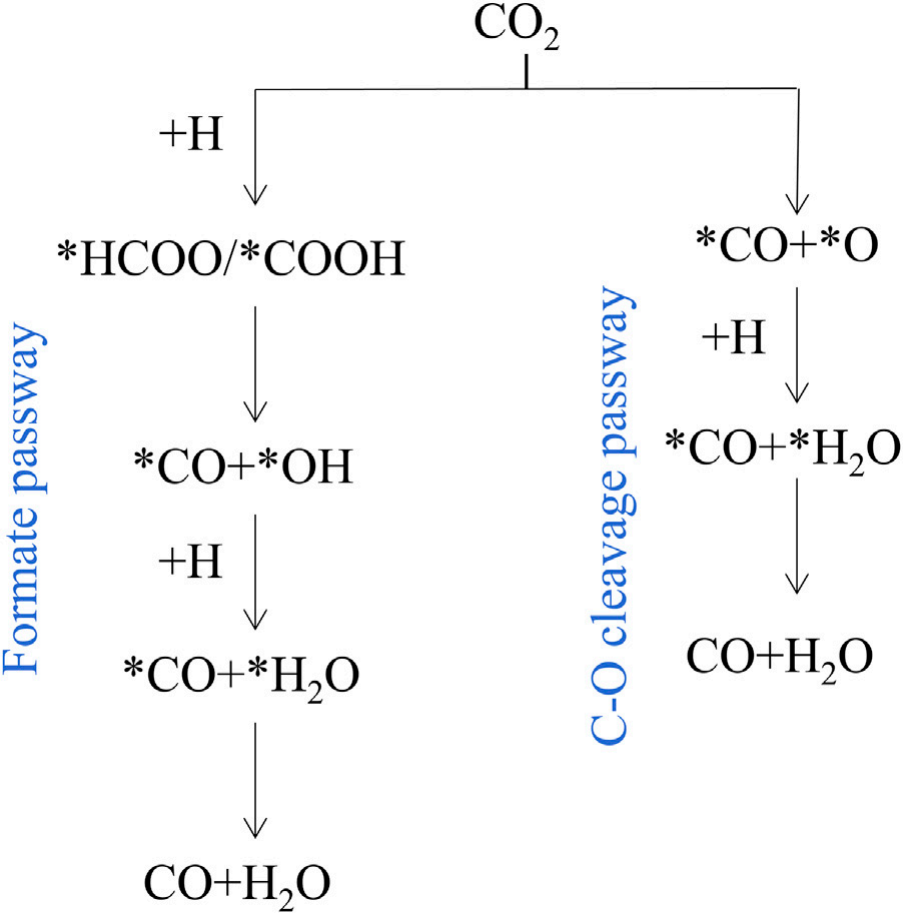
Simplified RWGS mechanism. Reproduced from Kattel et al. (2017); Copyright ^©^ 2017 (American Chemical Society).

RWGS reaction on catalysts mainly proceeds through the redox mechanism or the formate dissociation progress (Chen et al., 2020). As shown in Figure 2, there are two main reaction pathways reported in literature 1) Formate pathway: goes on via more reactive carboxyl (*COOH) or formate (*HCOO) intermediates; 2) C-O bond cleavage pathway: CO_2_ is directly decomposed into *CO and *O. In the metal oxide systems, the metals adsorb dissociative H_2_ and spill it to the M-O sites in which CO_2_ is adsorbed (Lindenthal et al., 2020). Typically, in perovskite oxide (ABO_3_), CO_2_ conversion occurs on oxygen vacancies, and oxygen-deficient structures (ABO_3-δ_) are formed by reduced H_2_ (Kopac et al., 2020). According to all the catalysts reported so far, it is indicated that both mechanisms are common in any reaction, and which route has a relative advantage over the other depends on the specific catalyst (Thalinger et al., 2015).

**FIGURE 3:**
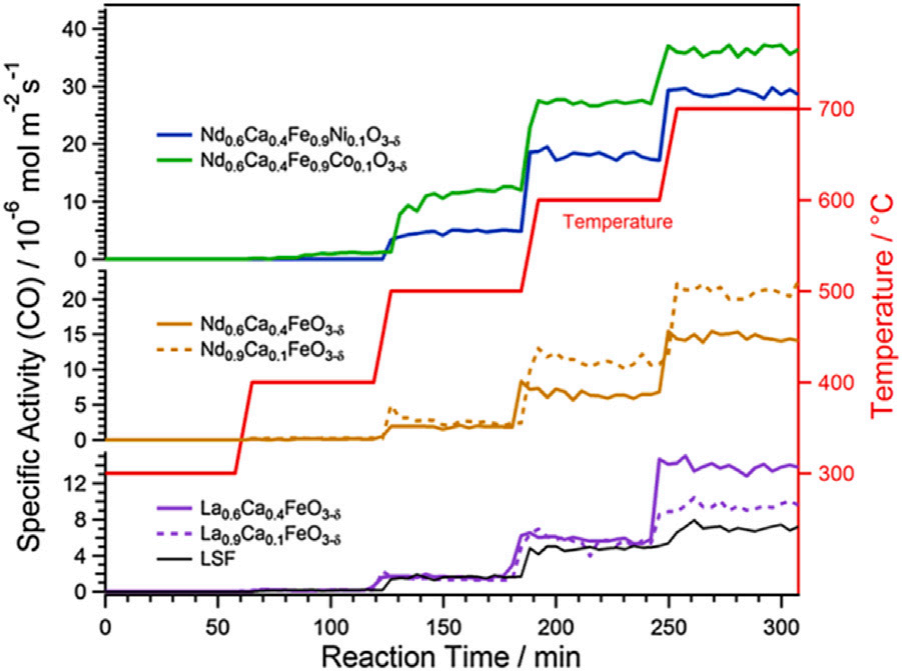
Comparison of RWGS reaction activity results on Co-, Ca-, and Ni-doped samples. Reproduced from Lindenthal et al. (2021); Copyright ^©^ 2021 (Elsevier).

Considering the importance of oxygen vacancies in the RWGS reaction, perovskite-like structure materials with high content of oxygen vacancies have been promising candidates (Maiti et al., 2018). Perovskite oxides (ABO_3_) are readily doped with highly reactive elements, these dopants escape from the perovskite lattice or form nanoparticles through diffusion (by exsolution) after controlled reduction or during the reaction, which leads to more oxygen vacancies generated thereby increasing the performance of RWGS reaction (Lindenthal et al., 2020). On the other hand, the exsolution of active metal well dispersed on perovskite surface, which is very beneficial to improve the catalytic activity, an example is shown in Figure 3 (Lindenthal et al., 2021). Analogously, Kuhn et al. synthesized five various Sr-doped lanthanum cobaltates, La_1-X_Sr_X_CoO_3−δ_ (0 ≤ X ≤ 1, with a step size of 0.25), to evaluate their carbon dioxide conversion properties. The result indicated that when X was 0.25, the La_0.75_Sr_0.25_CoO_3−δ_ sample carried the best structure stability under reducing conditions and the top CO generation ability during the CO_2_ reoxidation process (Daza et al., 2014). Meanwhile, they also found the strontium-doped La_0.75_Sr_0.25_FeO_3_ (LSF) perovskite-type oxide combined with silica promoted a prominent extent of oxygen vacancies in the active phase, and concomitant with a decreased average LSF crystallite size, resulting in unprecedented rates of reverse water gas shift chemical looping. Furthermore, the support SiO_2_ could also suppress the perovskite sintering through the interfacial wettability effect which is confirmed by visual examination of microscopy. The inhibiting species of FeSiO_3_ and La_2_SiO_5_, which may lead to interfacial energy barriers and thereby limit accessibility to active surfaces, can restrict its formation by adjusting the mass ratio of perovskite and support silica (Hare et al., 2018). The combination of different supports and different morphologies of perovskites produces unexpected outcomes, which in turn exhibit different RWGS properties. For example, the researchers also developed the effect of using various supports (CeO_2_, Al_2_O_3_, SiO_2_, TiO_2_, and ZrO_2_) in combination with perovskite oxides for RWGS (Hare et al., 2018; Hare et al., 2019b). It is worth noting that the synthesis method of perovskite also influences the RWGS performance (Lim et al., 2021b; Jo et al., 2022).

**FIGURE 4:**
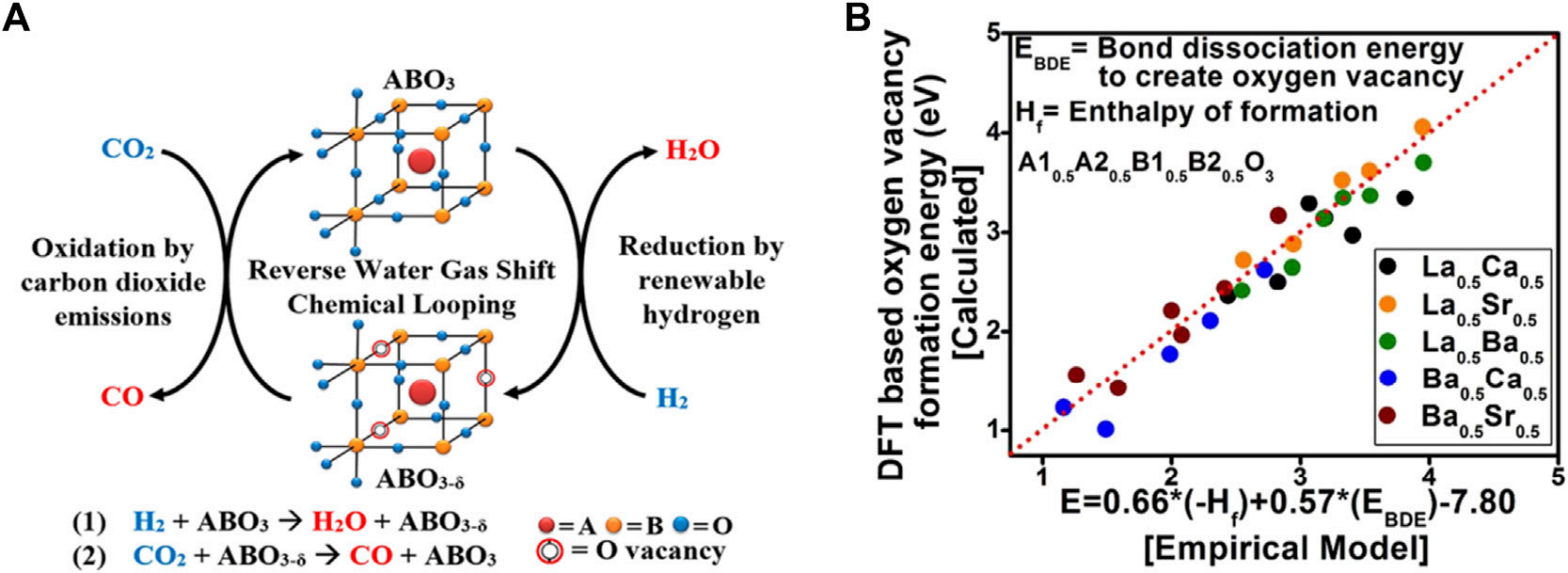
The process of RWGS-CL **(A)** Reproduced from (Hare et al., 2018); Copyright ^©^ 2018 (American Chemical Society) and the empirical modeling of oxygen vacancy formation energies (Evac) of the perovskite oxides **(B)** Reproduced from Maiti et al. (2018); Copyright ^©^2018 (Royal Society of Chemistry).

The production of carbon monoxide via conventional, thermally driven RWGS is a costly process, requiring energy-intensive operating conditions. To decrease the operating temperature, Kawi et al*.* used non-thermal plasma (NTP) combine with perovskite La_0.9_Ce_0.1_B_0.5_B′_0.5_O_3−δ_-derived bimetallic catalysts (B: Cu, Ni, Fe, B’: Ni, Fe, Cu) formed a dielectric barrier discharge plasma-catalysis system to ignite RWGS reaction, the results revealed that the plasma-catalysis system has excellent capability to promote the RWGS reaction at low temperature and normal pressure (Liu et al., 2020b; Liu et al. 2020b; Liu et al. 2022). Furthermore, RWGS reaction with chemical looping (RWGS-CL) (Maiti et al., 2018; Lim et al., 2021b; Lee et al., 2022), which is comprised of a two-step redox step: reduction procedure by renewable H_2_ and oxidation step by CO_2_, would be a promising method because it can considerably reduce the operating temperature of the reduction process. High oxygen mobility of the perovskite oxides allows for the operation of these looping cycles without phase change of the oxides. The process is depicted in Figure 4A. The mechanism of RWGS-CL mainly relies on the generation of oxygen vacancies on these surfaces and the conversion of carbon dioxide to these oxygen vacancies. Therefore, probing these oxygen vacancies on different perovskite oxide compositions is essential to better formulate catalysts and understand their roles in CO_2_ conversion. Bhethanabotla et al. using density functional theory (DFT) calculated the oxygen vacancy formation energy in different perovskite oxides during CO_2_ conversion reaction (Figure 4B), and they found that using lanthanum and Ca-based perovskite oxides can achieve 100% selective CO generation at record low-temperature process temperatures of 450–500°C, and these materials performed very stably in several RWGS-CL cycles (Maiti et al., 2018).

### CO_2_ methanation to CH_4_

The CO_2_ methanation, also known as “Sabatier reaction”, was discovered by Sabatier et al., in 1902 (Senderens and Sabatier, 1902). From a thermodynamics perspective (Ye et al., 2019b), the enthalpy and Gibbs free energy of the CO_2_ methanation process are both negative, indicating a very favorable process (Eq. (2)) (Tsiotsias et al., 2020).

$$CO_{2}+4H_{2}\to CH_{4}\mathrm{+2}H_{2}\mathrm{O,}\mathrm{\Delta Η=-165}\;\mathrm{kJ}/\mathrm{mol}\mathrm{\&\Delta G=-130}\mathrm{.8}\;\mathrm{kJ}/\mathrm{mol}$$ (2)

Although thermodynamically favored, the reaction is kinetically limited due to the high inertness of CO_2_. Indeed experimental CO_2_ methanation does not yield significant methane production at room temperature and atmospheric conditions (González-Castaño et al., 2021). Therefore, many studies have been carried out on CO_2_ methanation to CH_4_ in various types of catalytic systems (Wang et al., 2016; Rosid et al., 2019; Lv et al., 2020; Pastor-Pérez et al., 2020). However, CO_2_ methanation catalysts are prone to rapid and severe deactivation during the reaction process due to various physicochemical changes such as thermal degradation of support materials, metal sintering, and especially coke formation (le Saché et al., 2020; Sreedhar et al., 2019). Therefore, the development of effective and stable catalysts lefts a major challenge for CO_2_ methanation commercialization (Mebrahtu et al., 2018; Ashok et al., 2020).

**FIGURE 5:**
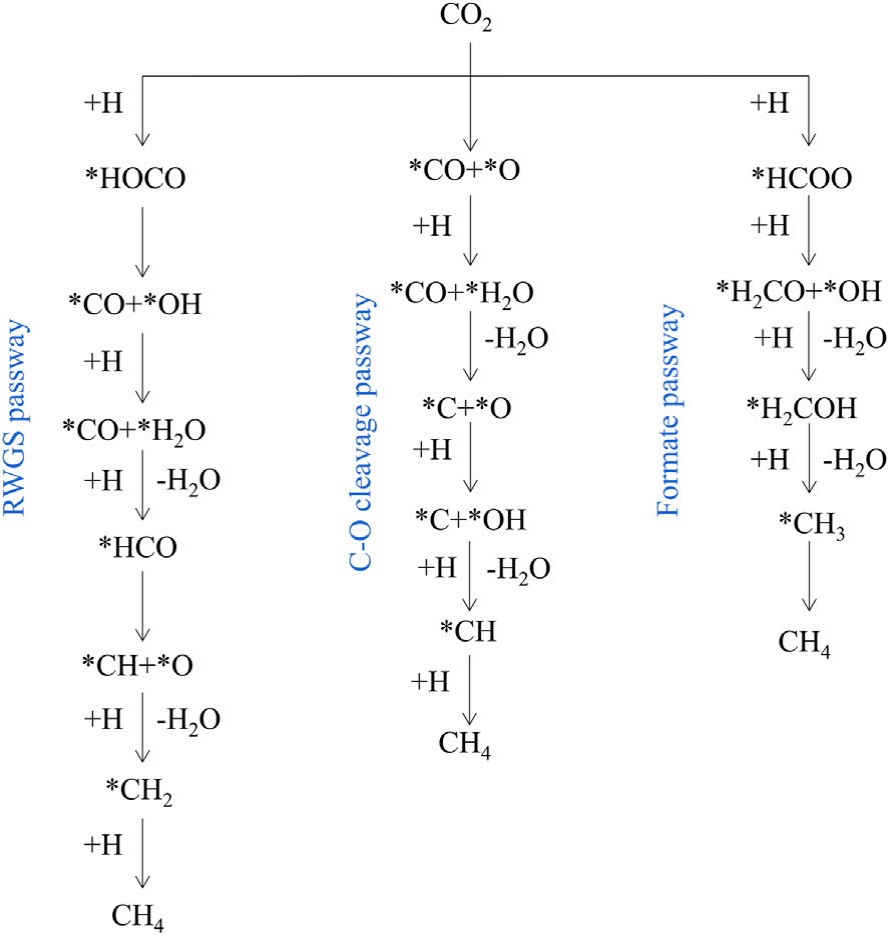
Simplified CO_2_ methanation reaction mechanism. Reproduced from Kattel et al. (2017); Copyright ^©^ 2017 (American Chemical Society).

In order to rationally design advanced catalytic systems, it is necessary to study the reaction mechanism of CO_2_ methanation (Lv et al., 2020). Roughly there are three potential reaction pathways well-accepted in literature: 1) RWGS pathway: Proceeds through *CO and then undergoes consecutive *CO hydrogenation via *HCO which ends up in *CH_x_ species to produce methane. 2) C-O bond cleavage pathway: proceeds through direct dissociation of CO_2_ generates *CO and *O, and then *CO is further dissociated to *C and *O, the *C is hydrogenated to methane. 3) Formate pathway: proceeds through *HCOO and then consecutive hydrogenation via *H_2_CO and *H_2_COH which end up in *CH_3_ species to produce methane (Figure 5) (Aziz et al., 2015; Roy et al., 2018; Hussain et al., 2021). Typically, the presence of multiple active sites on the catalyst surface promotes the activation and dissociation of reactants to generate the desired product through different reaction intermediates (Rosid et al., 2019; Sreedhar et al., 2019; Riani et al., 2021).

The CO_2_ methanation reaction system is often accompanied by complex multiple side reactions, and the formation of coke from the side reactions is the main reason for the CO_2_ methanation catalysts' deactivation (Hussain et al., 2021). Another main CO_2_ methanation catalysts drawbacks concerning deactivation issues are high-temperature sintering (Roy et al., 2018). Therefore, the development of anti-carbon deposition and high-temperature sintering resistance catalysts is the key to solving the bottleneck of CO_2_ methanation (Ashok et al., 2020; Price et al., 2021). During the CO_2_ methanation reaction, oxygen vacancies usually play a role in favoring CO_2_ adsorption and enhancing the ability to resist carbon deposition (González-Castaño et al., 2021; Blanco et al., 2022). In addition, the oxygen vacancies are also considered to be the key factor for C-O dissociation obtaining higher CH_4_ yields (Wang et al., 2016). Considering the importance of oxygen vacancies, perovskite-like materials are promising candidates due to their the elevated oxygen mobility and high-temperature stability (González-Castaño et al., 2021). The generation, recovery, and regeneration of oxygen vacancies (cycle process) are often accompanied by the occurrence of redox reactions (Zhang J et al., 2021). It is found that the using (or doping) of variable valence metal in perovskite (A/B site) can accelerate the generation of oxygen vacancies or elevated oxygen mobility in the CO_2_ methanation (Li S et al., 2020; Zhang J et al., 2021). For example, M. González-Castaño et al. synthesized Ni catalyst supported on YMnO_3_ perovskite via coprecipitation method, and the replacement of Mn^3+^ by Ni^2+^ atoms result in the formation of Mn^4+^ species by way of a charge compensation mechanism, which attained the ability to exchange oxygen species, leading to the remarkable performance with TOFs = 20.1 s^−1^ at 400°C and 60 L/(g·h). The presence of oxygen vacancies in the YMnO_3-x_ support effectively enhances the dissociative adsorption of CO_2_ through easier redox interconversion, resulting in high activity and stable catalytic behavior without evidence of deactivation (González-Castaño et al., 2021). Similarly, the variable valence metals like Ce (Ren et al., 2021; Zhang J et al., 2021), Fe (Thalinger et al., 2016b; Steiger et al., 2020), Ti (Tang et al., 2018; Do et al., 2020; Jiang et al., 2021), etc. can also lead to oxygen vacancies increase in perovskite structure materials.

**FIGURE 6:**
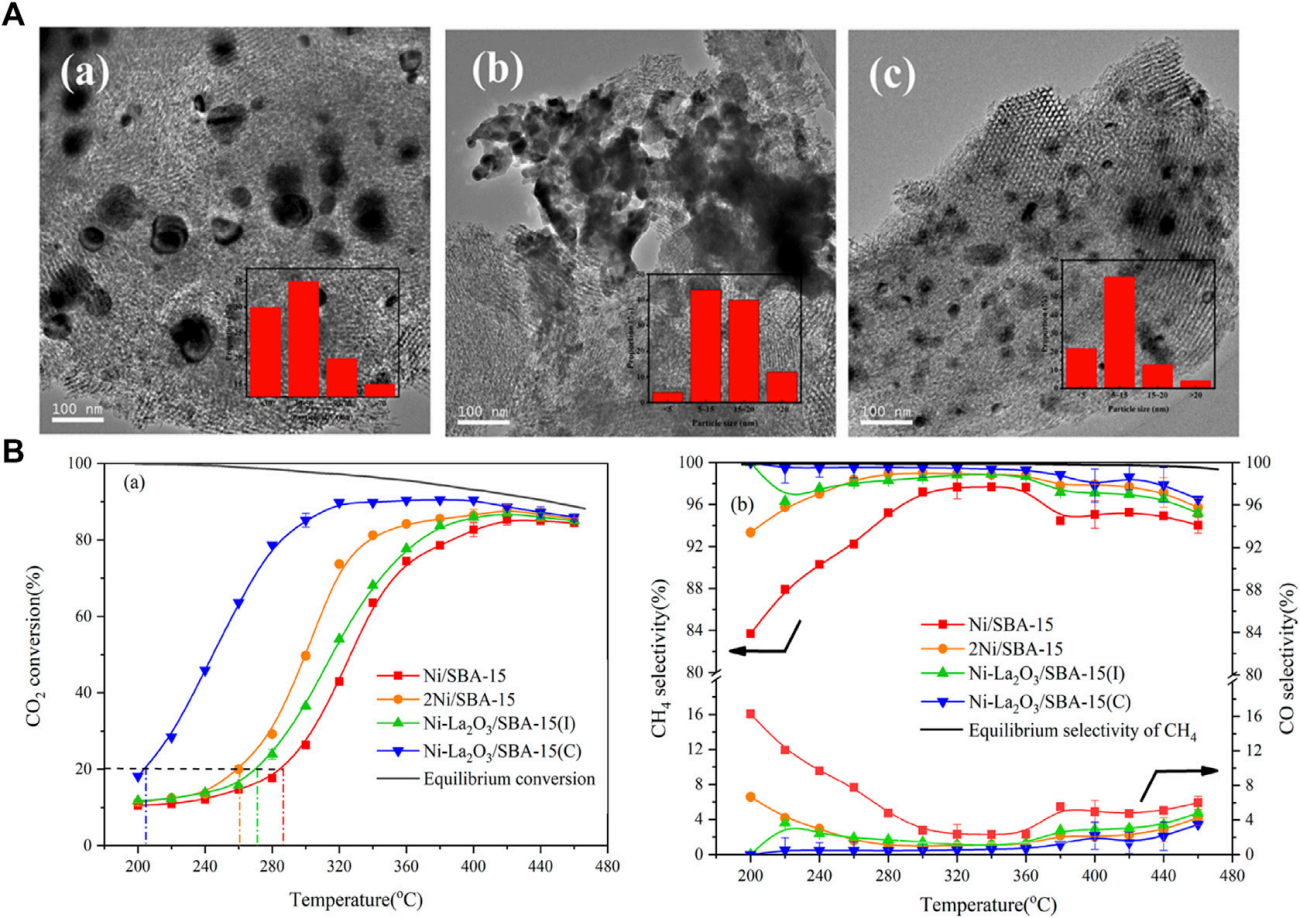
**(A)** TEM images for the catalysts after reaction: Ni/SBA-15 **(a)**; Ni-La_2_O_3_/SBA-15(I) **(b)**; Ni-La_2_O_3_/SBA-15**(c)**. **(B)** Catalytic activity and selectivity of the catalysts. Reproduced from Wang X et al. (2019); Copyright ^©^ 2019 (American Chemical Society).

It is found that the dispersion of active metals has a great influence on the performance of the CO_2_ methanation reaction (Lim et al., 2021a). In order to attain well-dispersed active metals, the metal loading on the catalyst is usually low to suppress agglomeration during the catalyst preparation. Low loading of active species would inevitably lead to relatively low activity; thus, the research focus is on fabrication catalysts with high dispersion under high loading (Jiang et al., 2021). In addition to oxygen mobility properties, perovskite oxides also exhibit good reactivity and thermal stability at higher metal loadings, so they are often used as redox catalysts in CO_2_ methanation reactions. Apart from increasing the dispersion of active metal by substitution (Lim et al., 2021a; Jiang et al., 2021), a variety of tandem catalysts consisting of two interfaces with a single structure has been recently designed and used to catalyze the continuous reaction of CO_2_ hydrogenation to methane (Do et al., 2020). Wang et al. utilize LaNiO_3_ with perovskite structure as a La-modified catalysts precursor to the synthesis of Ni-La_2_O_3_/SBA-15(C) for CO_2_ methanation. Owing to the LaNiO_3_ distinct perovskite structure, the interaction between La and Ni is enhanced, thereby reinforcing the synergistic effect of La_2_O_3_ and Ni, making Ni nanoparticles with high dispersibility as well as satisfactory resistance to sintering and carbon deposition. In addition, compared to the Ni-La_2_O_3_/SBA-15 catalyst synthesized by the traditional wet impregnation method, the Ni-La_2_O_3_/SBA-15(C) demonstrated a higher dispersion of Ni and displayed a better catalytic performance with a CO_2_ conversion of 90.7% and a CH_4_ selectivity of 99.5% at 320°C (Figure 6) (Wang X et al., 2019). Coincidentally, Liu G et al. (2020) also utilized the specific perovskite structure of LaNiO_3_/LaNi_1-x_Co_x_O_3_ to synthesize LaNi_1-x_Co_x_O_3_-based catalysts supported on mesostructured cellular foam (MCF) silica (LaNi_1-x_Co_x_O_3_/MCF) and evaluate its CO_2_ methanation performance. The highly dispersed La_2_O_3_ and Ni/Ni-co alloy nanoparticles were formed within the pores of the MCF support after reduction, which exhibited high performance (Zhang and Liu 2020a; Zhang and Liu 2020b). In addition to the above SBA-15 and MCF, using perovskite-structured LaNiO_3_ as the precursor, a high-performance CO_2_ methanation catalyst with highly dispersed Ni nanoparticles and strong metal interactions was prepared, which also appeared on the supports of CeO_2_ (Onrubia-Calvo et al., 2021), ZrO_2_ (Li S et al., 2020), SiO_2_ (Li S et al., 2019), *γ*-Al_2_O_3_ (Do et al., 2020) etc.

To avoid the agglomeration of active metal, it is desirable to have strong SMSI between the metal and support to achieve high performance (Shin et al., 2016; González-Castaño et al., 2021). Shin et al. study found that at the same loading of Co. and Pt (1 and 0.2 wt%, respectively), the barium zirconate support provides a more than six-fold increase in CH_4_ formation rate, accompanied by a high CH_4_ selectivity as compared to previously studied *γ*-Al_2_O_3_ supports at 325 °C. This enhancement is attributed to a strong interaction between the Co. particles and the BaZrO_3_ support, as well as atomically dispersing of the Pt. It is noted that Pt atoms decorating the surface Co/CoO_x_ interface within the nanoparticle, and prefers to remain associated with the metallic Co. core as opposed to being incorporated into the CoO_x_ shell during oxidation of the particle (Shin et al., 2016). However, Penner, et al*.* reported that strong metal-support interactions have limitations in complex metal-oxide systems. They employed Rh/Ni-La_0.6_Sr_0.4_FeO_3-δ_ and Rh/Ni-SrTi_0.7_Fe_0.3_O_3-δ_ as precursors to explore the relationship between metal-support interactions and the performance of CO_2_ methanation. The results exhibited there is no typical, reversible strong metal-support interaction during the reaction (Thalinger et al., 2016a; Thalinger et al., 2016b).

**FIGURE 7:**
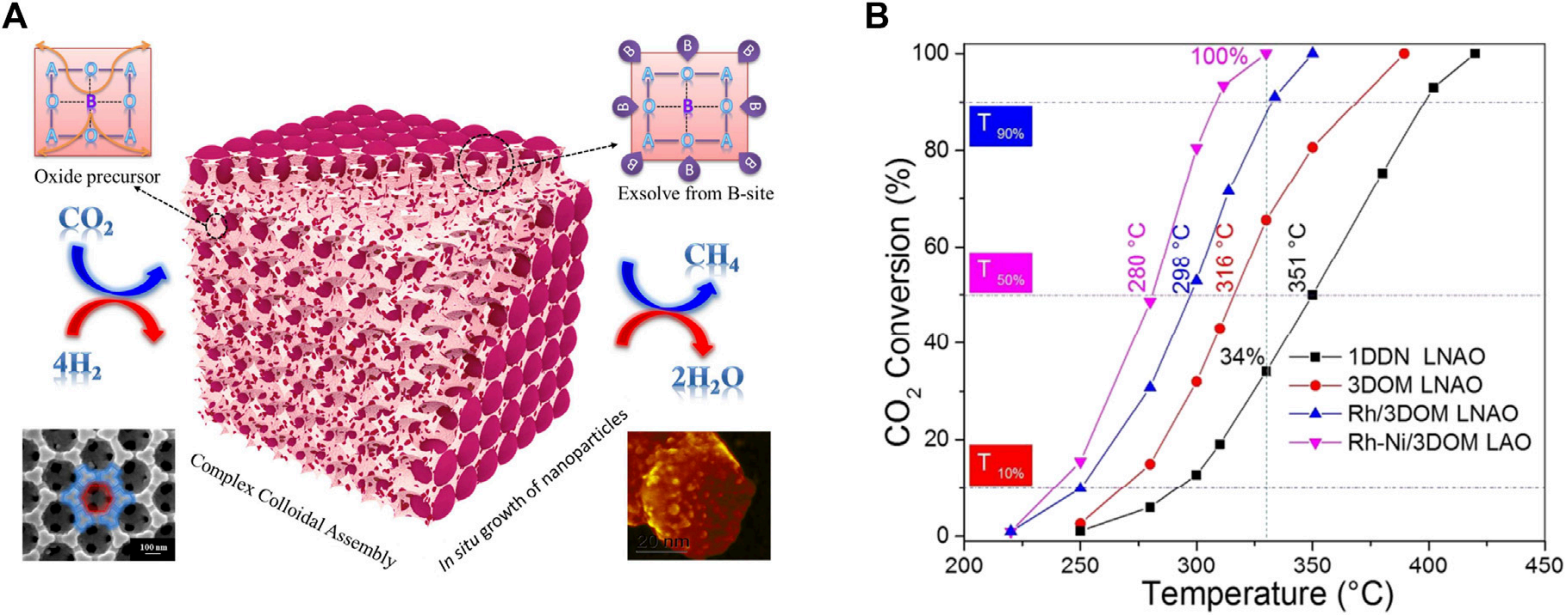
Schematic illustration of *in situ* exsolution of the catalyst from an ABO_3_ perovskite structure **(A)**, and catalytic activities for the different catalysts **(B)**. Reproduced from Arandiyan et al. (2018); Copyright ^©^ 2018 (American Chemical Society).

In addition to regular doping modification or using supports, novel strategies like special preparation methods or materials have emerged to improve the performance of CO_2_ methanation over perovskite structure (Arandiyan et al., 2018; Wang X et al., 2019). The colloidal crystal template method is widely used to create three-dimensional ordered macroporous (3DOM) structured materials due to its single-step low-temperature process, facile control in composition and morphology, and wide applicability to metal precursors. The interconnected porous network of 3DOM structured materials enables to control the active metal particle size and dispersion and influences the metal-support interaction during the exsolution process. For example, Rose Amal *et.al* using a poly(methyl methacrylate) microsphere colloidal crystal-templating route successfully prepared Ni-Rh nanoalloy/3DOM LaAlO_3_, the schematic illustration of *in situ* exsolution of the catalyst from an ABO_3_ perovskite structure shown in Figure 7A. The reduced Ni-Rh/3DOM LaAlO_3_ has high dispersion of bimetallic Ni-Rh NPs, rich surface adsorbed oxygen species and basic sites, and strong metal-support interaction after reduction treatment. The performance of CO_2_ conversion confirmed a significant enhancement in activity for the RhNi/3DOM LaAlO_3_ sample relative to the other catalysts (Figure 7B) (Arandiyan et al., 2018). Similarly, Wang et al. also used a template of poly methyl methacrylate colloidal crystal to synthesize Ni/Y_2_Zr_2_O_7_-3DOM, which has a much stronger interaction of NiO and the Y_2_Zr_2_O_7_-3DOM than Y_2_Zr_2_O_7_-CP support synthesized by a co-precipitation method. Under reducing conditions, the strong interaction of NiO and the Y_2_Zr_2_O_7_-3DOM achieved high active Ni surface and large quantities of surface-active O_2_^−^/alkaline sites, which are the mainly active agent trigger the CO_2_ methanation (Fang et al., 2021). Besides, the water is generated because of hydrogen oxidation and RWGS side reactions during CO_2_ methanation, which may cause the catalyst deactivation for not stable in water materials. For this, Kageyama et al*.* investigated the perovskite-type oxyhydride BaTiO_2.4_H_0.6_ as an effective water-stable support material for Ni-, Ru-based catalysts for CO_2_ methanation. The result proved that the oxyhydride support is 2–7 times more active for Ni and Ru than the oxide support of BaTiO_3_ (Tang et al., 2018).

**FIGURE 8:**
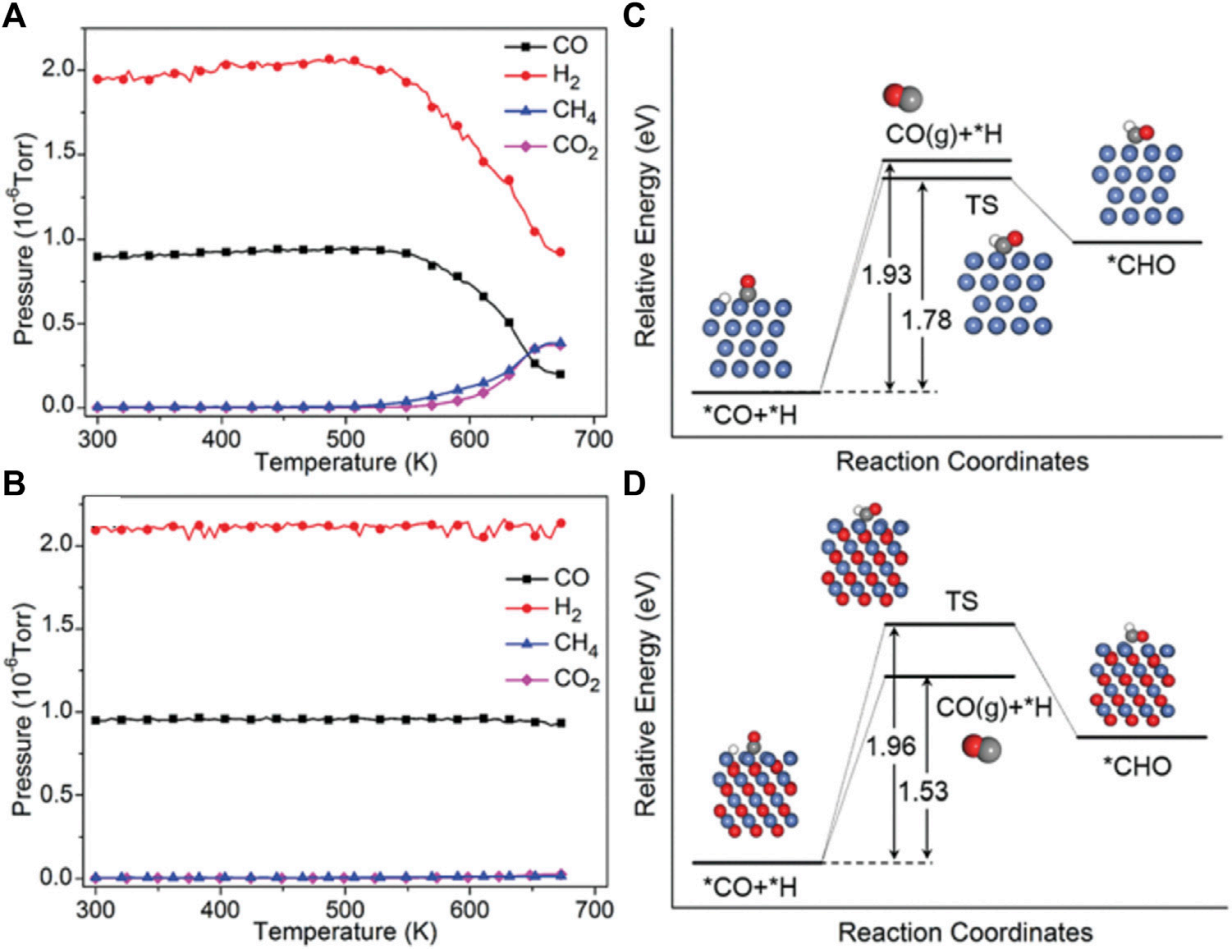
The partial pressures of CO, H_2_, CH_4_, and CO_2_ during the CO hydrogenation reaction for LaNiO_3_ **(A)** and LaFe_0.5_Ni_0.5_O_3_ **(B)**. Potential energy diagram for the reaction routes of *CO + H on Ni (111) **(C)** and NiO (111) **(D)**. Reproduced from Zhao et al. (2018); Copyright ^©^ 2018 (Royal Society of Chemistry).

According to the mechanisms of CO_2_ methanation, it can be found that there is one mechanism through consecutive RWGS and CO hydrogenation. That means the selectivity of CO_2_ hydrogenation to CH_4_/CO can be adjusted by controlling the stability of the CO intermediate. It is found that crystalline oxide catalyst shows good catalytically active in both CO_2_ methanation and the RWGS routes and has different selectivity and activity under various reaction conditions (Tsiotsias et al., 2020). The products can be selectively controlled by adjusting the reaction temperature or the type of catalyst (Ma et al., 2019; Ho et al., 2020; Xu et al., 2022). For example, Chen et al*.* found that by changing the valence state of Ni, the product selectivity of CO_2_ hydrogenation can be adjusted on La-Fe-Ni perovskites, and the result suggests that higher-valence nickel-related species could produce more CO. They analyzed technology to illustrate that under reaction conditions, metallic Ni and higher-valence nickel-related species were formed on LaNiO_3_ and LaFe_0.5_Ni_0.5_O_3_, respectively. Furthermore, the DFT calculations indicate that CO is weakly bound to NiO (111), and the desorption of *CO is more favorable than its further hydrogenation to CH_4_, resulting in higher selectivity for CO (Figure 8) (Zhao et al., 2018). Similarly, Scott et al*.* have studied the product selectivity of CO_2_ hydrogenation by K cation substitution of La over LaNiO_3_ perovskite catalysts. It is found that when the potassium incorporation is up to 0.2, the La_0.8_K_0.2_NiO_3_ have the maximum amount of NiO in the catalyst which leads to an increase the CO selectivity. Therefore, keeping nickel-related species in higher-valence states under the reaction conditions is one of the important strategies in promoting CO selectivity (Tsounis et al., 2020).

### CO_2_ reforming of CH_4_

The greenhouse gases of CH_4_ and CO_2_ are major contributors to global warming. The conversion of CH_4_ and CO_2_ to syngas (H_2_ + CO) has plentiful applications in synthetic chemistry (Li et al., 2021). Therefore, CO_2_ reforming of CH_4_ can not only alleviate global environmental problems but also provide a valuable chemical feedstock (Monteiro et al., 2019). It has been proved that the reserves of combustible ice (Gas Hydrate/Natural Gas Hydrate) in the South China Sea are as high as about 200 million cubic meters, equivalent to eight million tons of oil. Among many mining methods, the CO_2_ replacement method is a new mining method of combustible ice, which inevitably causes natural gas contaminated with CO_2_ in the product gas. Therefore, the efficient utilization of methane, especially the reforming of carbon dioxide, has attracted widespread attention. Simultaneously, the greenhouse gases (GHG) methane and carbon dioxide are the main “culprits” of global warming, their efficient use has always been a research focus (Wang et al., 2016; Wu et al., 2020).

CO_2_ reforming of CH_4_ is also called dry reforming of CH_4_ (DRM) due to not involving water in reactants, and it is an extremely endothermic reaction (Eq. (3)) (Abdullah et al., 2017). Therefore, it needs exceedingly high temperatures to achieve high equilibrium conversion of syngas at which supported metal catalysts are easily deactivated by sintering (le Saché and Reina 2022). Although the DRM produces H_2_/CO ratio with one theoretically, the simultaneous occurrence of side reactions of RWGS, CH_4_ decomposition (MD: Eq. 4), and the Boudouard reaction (BR: Eq. 5) causes the H_2_/CO ratio not close to one (Pakhare and Spivey 2014). Apart from affecting the ratio of synthesis gas, the occurrence of side reactions of the MD or BR can also lead to carbon deposition. Therefore, it is necessary to build up a thermally stable catalyst to suppress carbon deposition and sintering (Liu Y et al., 2020).

$$CO_{2}+CH_{4}\to 2\mathrm{CO+2}H_{2},\mathrm{\Delta Η=+247}\;\mathrm{kJ}\;\mathrm{mo}l^{\mathrm{-1}}$$ (3)

$$CH_{4}\to C\left(s\right)\mathrm{+2}H_{2},\mathrm{\Delta Η=+75}\;\mathrm{kJ}\;\mathrm{mo}l^{\mathrm{-1}}$$ (4)

$$\mathrm{2CO}\to C\left(s\right)\mathrm{+C}O_{2},\mathrm{\Delta Η=-171}\;\mathrm{kJ}\;\mathrm{mo}l^{\mathrm{-1}}$$ (5)

Typically, CH_4_ is activated on metals such as Rh, Pt, and Ni to produce carbon, CH_x,_ or formyl intermediates, while CO_2_ is activated at the support or interface of the catalyst to form carbonate precursors (Wang et al., 2016; Li et al., 2021). During the DRM reaction, the reduction of CO_2_ to CO is accompanied by the generation of oxygen-containing species (or oxygen vacancies) and the enhancement of oxygen mobility, which is beneficial to the oxidation of surface carbon formed by CH_4_ activation, thereby eliminating carbon deposition (Monteiro et al., 2019). Based on this, the high oxygen mobility exhibited by perovskite-like materials makes them promissory candidates applied in DRM reactions (Bian et al., 2020; Bhattar et al., 2021). Besides, the high-temperature stability of perovskite-like materials further exacerbates their exploitation in DRM reactions (Shi et al., 2021). In general, LaNiO_3_ with perovskite’s structure is widely studied, which is usually decomposed to the Ni/La_2_O_3_ catalyst after H_2_ activation or DRM reaction. Over the LaNiO_3_ perovskites, the presumed mechanism is the adsorption of methane on metallic nickel particles and the subsequent cracking to form carbon deposits, which is recognized as the rate-determining step. At the same time, CO_2_ reacts with La_2_O_3_ to generate La_2_O_2_CO_3_ intermediate, which then reacts with carbon to form CO at the Ni^0^-La_2_O_2_CO_3_ interface accompanied by the recovery of Ni metal surface (Gallego et al., 2008; Moradi et al., 2010; Sadykov et al., 2013).

**FIGURE 9:**
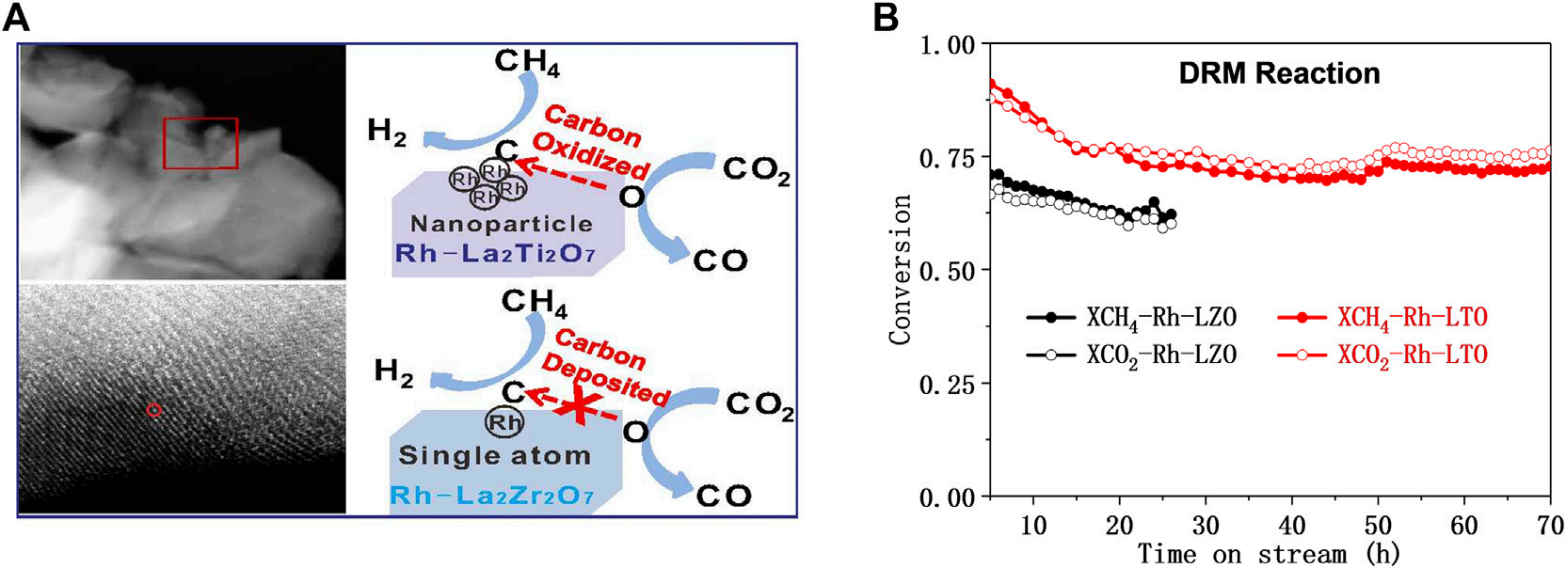
Schematic diagram of the DRM reaction **(A)** and the lifetime test on Rh substituted-La_2_B_2_O_7_ (B = Zr or Ti) **(B)**. Reproduced from Wu et al. (2018); Copyright ^©^ 2018 (American Chemical Society).

The use of hydrogen for pretreatment to obtain catalytically active metal oxide materials before DRM catalysis is still the preferred preparation method for promising perovskite-based DRM catalysts (Gallego et al., 2006). For example, the Rh substituted-La_2_Zr_2_O_7_ (pyrochlore-type) and La_2_Ti_2_O_7_ (perovskite-type) performed different DRM catalytic performances (Wu et al., 2018). Under reducing conditions, almost all Rh species substituted Zr made reactive oxygen species difficult to transfer, leading to the depositing of intermediate carbon on Rh-La_2_Zr_2_O_7_. On the contrast, part of Rh substituted Ti on Rh-La_2_Ti_2_O_7_ obtained coexistence of Rh^0^ and Rh^δ+^ after H_2_ was reduced, which accelerates the mobility of active O* and leading excellent activity and long-term stability for DRM (Figure 9). Therefore, the morphology and structural stability of perovskite-type mixed oxides-based materials also have a great impact on the DRM performance (Ho et al., 2020). Batiot-Dupeyrat et al. compared the La_2_NiO_4_ and LaNiO_3_ perovskite to be a precursor to exploring the performance of DRM. They found that after reduction treatment, La_2_NiO_4_ has the smallest nickel particles, making its catalytic activity higher than that of Ni/La_2_O_3_ or LaNiO_3_ (Gallego et al., 2006; Nezhad et al., 2021).

In order to elevate the DRM activity and stability of perovskite structure materials, the most popular method is to do a part substitution. The most common substitution metals can be divided into alkaline earth metals (Mg, Ca, Sr, Ba et al.) (Dama et al., 2018; Bekheet et al., 2021), rare earth and variable valence metals (Sm, Ce, Nd, Gd, Cu, Mn, Cr et al.) (Moradi et al., 2012; Bhavani et al., 2013; Wang et al., 2018), VIII group (Fe, Co., Ni, Ru, Rh, Pd, Ir) (Wang H et al., 2019; Das et al., 2020; Managutti et al., 2021), and others (Moradi et al., 2014). The alkaline earth metals substitution perovskite always increases the basicity, the strong exsolved Ni particle/support interfacial interaction thereby the DRM catalytic activity (Yang et al., 2015; Wei et al., 2020). In the DRM redox reaction system, we usually replace a proper amount of site A/B with Ce (or Mn, Cu, etc. variable valence metal) in the mixed-oxide. Then the Ce^3+^/Ce^4+^ (or Mn^2+^/Mn^3+^/Mn^4+^, etc.) cations can reversibly shuttle between mixed-oxide and CeO_2_, which enhanced the oxygen vacancies or oxygen mobility and thereby improved the catalytic activity (Wang et al., 2018). The VIII group metals are usually an active site for CH_4_ decomposition. Thus, an appropriate substitution amount not only enhances the active metal exsolution but also improves the dispersion of active metal (Goldwasser et al., 2005; Oh et al., 2019). Furthermore, it is found that the use of silica materials (SBA-15, SiO_2_, etc.) (Rivas et al., 2010; Wang et al., 2013), SiC (Zhang Z et al., 2021), CeSiO_2_ (Rabelo-Neto et al., 2018), MgAl_2_O_4_ (Messaoudi et al., 2018), and Al_2_O_3_ (Moradi et al., 2013) as supports plays a role as promoters in the physicochemical and catalytic properties of the perovskite catalyst, especially the relatively high surface area of support promotes a highly dispersed and catalytic activity.

**FIGURE 10:**
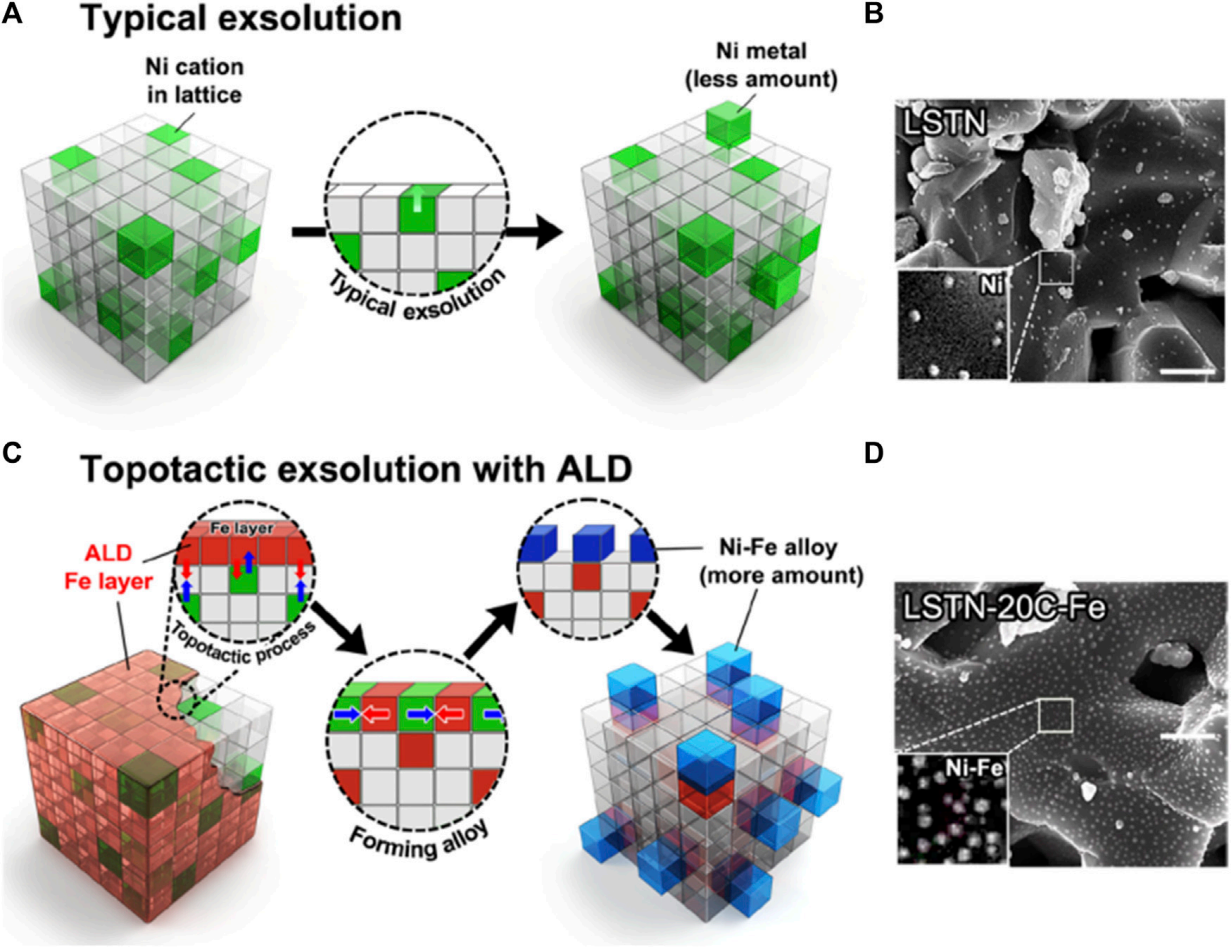
**(A)** Conventional exsolution for LSTN and **(B)** corresponding SEM image of LSTN. Scale bar, 500 nm. **(C)** Topotactic exsolution via ALD for LSTN-20C-Fe and **(D)** corresponding SEM image of LSTN-20C-Fe after reduction. Scale bar, 500 nm. Reproduced from Joo et al. (2020); Copyright ^©^ 2020 (Science).

In addition to the commonly used Pechini sol-gel method to prepare perovskite structural materials, co-precipitation and impregnation method have also been widely studied, but their development is limited due to obtained smaller specific surface area of the catalyst and unsatisfactory activity on DRM (Rivas et al., 2008; Yadav and Das 2019; Yafarova et al., 2019). Therefore, new synthetic methods have emerged. For example, Joo et al*.* used atomic layer deposition (ALD)-combined topotactic exsolution method to obtain Ni-Fe alloy (Joo et al., 2020), in which raw materials are La_0.6_Sr_0.2_Ti_0.85_Ni_0.15_O_3-δ_ and Fe_2_O_3_. The lower Ni-Fe alloy formation energy (-0.43 eV) enhanced the catalytic activity of DRM, prolonging its stability to 410 h (Joo et al., 2020; Ma et al., 2022). Figure 10 shows the process of conventional and topotactic exsolution via ALD. Other novel synthesis methods like the template method using SBA-15 as templating agent (Nair et al., 2014; Duan et al., 2017), ultrasonic spray pyrolysis method (Pereñíguez et al., 2010; Shahnazi and Firoozi 2021), microwave-assisted (Figueredo et al., 2018; Gangurde et al., 2018; Marin et al., 2021), magnetic distilled water-assisted (Mousavi and Nakhaei Pour, 2019; Mousavi et al., 2020), auto-combustion methods (Caprariis et al., 2015; Ruocco et al., 2019), one-step polymerization method (Silva et al., 2019) have also been performed in the preparation of efficient DRM catalysts. These newly developed synthesis methods could serve as a general powerhouse in other fields of energy utilization. In addition to improving the DRM activity from the perspective of catalysts, researchers have also tried to improve the performance of the reaction equipment and reaction conditions, such as using a plasma-assisted replaced heat source (Zheng et al., 2015), coupling chemical looping reforming, or autothermal reforming (Sastre et al., 2019).

## Conclusions and perspectives

The increased amount of CO_2_ in the atmosphere mainly due to the excessive consumption of fossil fuels plays a major role in climate changes on a global scale. Therefore, it is mandatory to reduce CO_2_ emissions and develop CO_2_ capture as well as CO_2_ utilization technologies. The conversion and utilization of waste CO_2_ emissions into value-added products, such as chemicals, fuels, and other materials, while restraining climate change has drawn attention, which is crucial for a sustainable future.

Considering the high oxidation and thermodynamic stability of CO_2_, various strategies such as the catalyst preparation method, preparation conditions, and the component, as well the reaction conditions, technical approaches have been exploited in the conversion of CO_2_. In this review, we particularly elaborate on the perovskite-type mixed oxides-based catalysts on DRM, CO_2_ methanation, and RWGS reaction. All these gas-phase CO_2_ conversion processes are considered direct routes for CO_2_ valorization. The bottleneck for their implementation at the commercial scale is the lack of a robust and selective catalyst that can deliver the desired products satisfying the energy demands and favoring an economically viable chemical process. Herein perovskite catalysts emerge as fairly promising materials. given their defects chemistry with a significant concentration of oxygen vacancies and high-temperature stability characteristic of perovskite structure. Furthermore, the improved performance of the conversion of CO_2_ on perovskite-type mixed oxides-based catalysts by site A/B substitution, novel preparation method, combined with supports, etc., have been summarized. Apart from catalyst design, technical approaches involving innovative reactors and new processes design such as combined non-thermal plasma, light-drive, thermo-electric, etc., are also applied to improve CO_2_ conversion. Although it would take some time to bring these technologies up to the levels of practical CO_2_ hydrogenation, society’s need for effective measures is driving these rapid advances to reduce the acceleration in global warming caused by growing CO_2_ emissions.

For the future research in this field, we have proposed several perspectives as follows: 1) a more advanced preparation method should be developed for the perovskite-type mixed oxides based catalysts; 2) The relationship between the structure and catalytic performance over perovskite-type mixed oxides based catalysts for CO_2_ conversions should be investigated by the *in-situ/operando* characterization and DFT computational methods. The reaction mechanism of CO_2_ conversions is still challenging as the structure of perovskite-type mixed oxides based materials is complicated and the reaction pathway is diverse; 3) The combination of the perovskite-type mixed oxides based catalysts with other kinds of materials such as metal-organic frameworks, layered double hydroxide, and carbon materials could also be investigated to further enhance the catalytic performance for CO_2_ conversions; 4) Taking advantage of the optoelectronic properties of some perovskite-type mixed oxides based materials, future research could introduce solar-energy to drive catalysts for higher CO_2_ conversion efficiency; 5) Considering the remarkable oxygen mobility and redox cycle ability of perovskite-type mixed oxides based catalysts, the future reaction system could combine multiple technologies such as chemical looping or integrated reactor systems such as membrane reactors favoring one-step reaction and separation and leading to process intensification. All in all, these new technologies shall pursue the sustainable synthesis of added value products using CO_2_ as a carbon pool at high conversion with minimal energy consumption paving the way toward a net-zero modern society.
